# The rodent malaria liver stage survives in the rapamycin-induced autophagosome of infected Hepa1–6 cells

**DOI:** 10.1038/srep38170

**Published:** 2016-11-30

**Authors:** Chenghao Zhao, Taiping Liu, Taoli Zhou, Yong Fu, Hong Zheng, Yan Ding, Kun Zhang, Wenyue Xu

**Affiliations:** 1 Department of Pathogenic Biology, Third Military Medical University, Chongqing, 400038, P. R. China; 2 Department of Microbiology, Third Military Medical University, Chongqing, 400038, P. R. China

## Abstract

It has been reported that non-selective autophagy of infected hepatocytes could facilitate the development of malaria in the liver stage, but the fate of parasites following selective autophagy of infected hepatocytes is still not very clear. Here, we confirmed that sporozoite infection can induce a selective autophagy-like process targeting EEFs (exo-erythrocytic forms) in Hepa1–6. Rapamycin treatment greatly enhanced this process in EEFs and non-selective autophagy of infected Hepa1-6 cells and enhanced the development of the malaria liver stage *in vivo*. Although rapamycin promoted the fusion of autophagosomes containing the malaria parasite with lysosomes, some parasites inside the autophagosome survived and replicated normally. Further study showed that the maturation of affected autolysosomes was greatly inhibited. Therefore, in addition to the previously described positive role of rapamycin-induced nonselective autophagy of hepatocytes, we provide evidence that the survival of EEFs in the autophagosome of the infected hepatocytes also contributes to rapamycin-enhanced development of the malaria liver stage, possibly due to the suppression of autolysosome maturation by EEFs. These data suggest that the inhibition of autolysosome maturation might be a novel escape strategy used by the malaria liver stage.

Malaria is one of the most devastating diseases worldwide. Approximately 3.4 billion people are at risk for malaria, and 207 million new cases with 627,000 deaths occur each year^1^. Malarial infection begins with the bite of a *Plasmodium*-infected mosquito. After they are injected into the host’s skin, sporozoites rapidly invade the liver and transform into EEFs (exo-erythrocytic forms) in hepatocytes. Mature schizonts are then released from the hepatocytes to invade red blood cells, initiating blood-stage infection. The clinical symptoms begin at this point, and patients are always clinically silent at the pre-erythrocytic stage. Thus, a better understanding of the mechanisms underlying the host-parasite interactions in the pre-erythrocytic stage could help in the design of improved prophylactic strategies.

During the pre-erythrocytic stage, malaria parasites are detected by the host and elicit robust innate immune responses. Our previous research has shown that sporozoites can be sensed by TLR2 and that the activation of TLR2 significantly suppresses intrahepatic parasite development^2^. *Plasmodium* RNA inside the hepatocyte is a PAMP (pathogen-associated molecular pattern) sensed by the PRR (cytosolic pattern recognition receptor) Mda5, inducing a type I IFN response^3^. The IFN-α/β released from the infected hepatocytes activates liver lymphocytes, including NK and NKT cells, to kill the parasite in hepatocytes through the secretion of IFN-γ^4^, ^5^, ^6^. In contrast, the pre-erythrocytic stage has evolved strategies to suppress or escape the host immune responses to facilitate development in the liver^7^. For example, sporozoite CSPs (circumsporozoites proteins) can inhibit the respiratory burst of Kupffer cells to facilitate their safe passage through these cells^8^. Pre-erythrocytic forms in the hepatocytes not only inhibit the apoptosis of infected hepatocytes but also translocate CSP from the parasitophorous vacuole into the cytoplasm to promote its development^9^,^10^. However, the details of interaction between the parasite and host cell are still largely unknown.

Macroautophagy, hereafter referred to as autophagy, is a bulk degradation system that delivers cytoplasm constituents and organelles into the lysosomes for hydrolysis. Autophagy can be selective or non-selective. Non-selective autophagy occurs in response to amino-acid deprivation and is essential for cell survival, development and homeostasis. Selective autophagy targets various targets, such as organelles and microorganisms, for degradation^11^. A growing body of evidence has shown that selective autophagy could restrict a variety of viral infections and the replication of intracellular bacteria and protozoa^11^. Some microorganisms have developed diverse strategies to subvert autophagy and, in some cases, utilize the components of autophagic pathways to facilitate their own replication^12^,^13^,^14^.

Very recently, it has been reported that liver-stage malaria infection can induce non-selective autophagy in infected hepatocytes and selective autophagy of the parasite by the host cell^15^,^16^. Although nonselective autophagy in the host cell was thought to be beneficial to parasite growth by providing nutrients, the fate of a parasite following selective autophagy of infected hepatocytes is not clear.

In this study, we found that *P. y. yoelii* sporozoites induce a selective autophagy-like process targeting EEFs, which can be greatly enhanced by rapamycin. Although rapamycin treatment can promote the fusion of autophagosomes containing parasites with lysosomes, some parasites survive inside the rapamycin-induced autophagosome. Then, we investigated the mechanisms underlying this phenomenon. We found that some EEFs survive and proliferate normally in autolysosomes. This phenomenon might be associated with their ability to inhibit the maturation of autolysosomes.

## Results

### Sporozoite infection induces a hepatic autophagy-like process targeting EEFs

**Figure 1:**
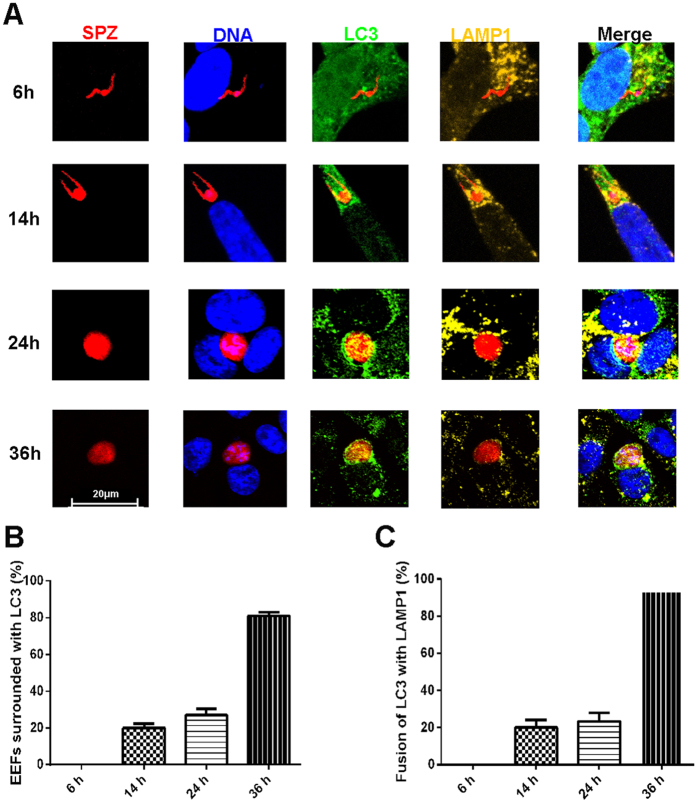
*P. y. yoelii* sporozoite infection induces a selective autophagy-like process targeting EEFs. After Hepa1-6 cells growing on a coverslip were incubated with *P. y. yoelii* 265BY-RFP sporozoites for the indicated time, the cells were stained with anti-LC3, anti-LAMP1 and DAPI and subjected to confocal microscopy. (**A**) A representative confocal microscopic image of EEFs in the autophagy-like vacuole at the indicated incubation time. (**B**) Percentage of EEFs in the autophagy-like vacuole at the indicated incubation time. (**C**) Percentage of LC3 and LAMP1 double-positive vesicles containing EEFs at the indicated time. All data are presented as the mean ± SD.

Although two papers have recently reported that infection with the *P. berghei* ANKA sporozoite can lead to host-cell autophagy of EEFs, but it is still unknown whether *P. y. yoelii* sporozoite infection can also induce autophagy of EEFs by hepatocytes. Therefore, the autophagy-specific protein LC3 (microtubule-associated protein 1, light chain 3)^17^ surrounding the parasite was examined after *P. y. yoelii* 265BY-RFP (Red fluorescence protein) sporozoites were incubated with Hepa1–6 *in vitro* for varying periods of time. Immunofluorescence analysis revealed that LC3 accumulation around the EEFs increases with the development of the liver stage. Punctuate LC3 could be found near the parasite, but no parasite was surrounded by the LC3 at 6 hpi (hours post infection). However, EEFs were completely encapsulated by LC3 after 14 hpi, and the intensity of LC3 increased gradually at 24 and 36 hpi (Fig. 1A), indicating a selective autophagy-like process targeting EEFs. Additionally, the percentage of EEFs in the autophagosome-like vacuoles increased from 20–27% at 14 and 24 hpi to more than 80% at 36 hpi (Fig. 1B).

A hallmark of autophagy is the fusion of LC3-positive autophagosomes with lysosomes. Thus, autophagosome-lysosome fusion was investigated by detecting the co-localisation of LC3 and LAMP1 around the EEFs. Again, the co-localisation of EEFs with the lysosomal marker LAMP1 was observed as early as 14 hpi and became more intense at 24, and 36 hpi (Fig. 1A). The percentage of EEFs in the autolysosome also increased from approximately 20% at 14 and 24 hpi to more than 90% at 36 hpi (Fig. 1C). These data suggest that sporozoite infection can induce selective autophagy-like events targeting EEFS in hepatocytes.

### Rapamycin promotes host cell a selective autophagy-like process targeting EEFs

**Figure 2:**
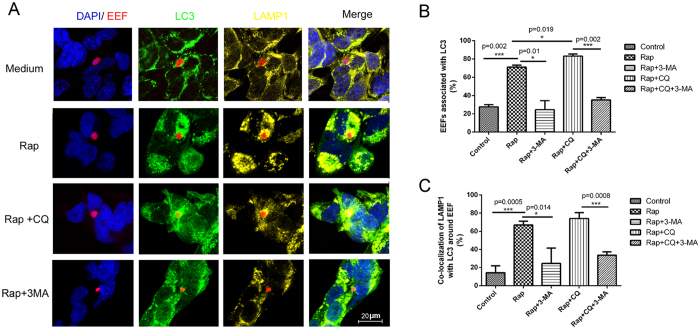
Rapamycin treatment promotes a hepatic autophagy-like process targeting EEFs. Hepa1-6 cells growing on a coverslip were incubated with *P. y. yoelii* 265BY-RFP sporozoites for 3 h. The cells were treated with the indicated stimulus for 24 h and then stained with anti-LC3 and anti-LAMP1, counterstained with DAPI, and subjected to confocal microscopy. (**A**) A representative confocal microscopy image of an autophagosome containing EEFs and the fusion of the autophagosome with a lysosome after incubation with the indicated compounds. (**B**) Statistical analysis of the autophagosomes containing EEFs. (**C**) Statistical analysis of the fusion of the autophagosomes containing EEFs with the lysosomes. One of three individual experiments is presented, and all data are presented as the mean ± SD. *P < *0.05*; ** *P* < *0.01.*

Rapamycin, the pharmacological inhibitor of mTOR (mammalian target of rapamycin), can be used to induce autophagy^18^. To investigate whether it can enhance autophagy of EEFs by hepatocytes, rapamycin was added at 3 h of incubation of sporozoites with Hepa1–6. At 24 hpi, the administration of rapamycin greatly increased the number of Hepa1-6 cells with large accumulations of LC3 (Fig. 2A), indicating the induction of non-selective autophagy in hepatocytes. Interestingly, punctate LC3 was also found surrounding the EEFs (Fig. 2A), indicating that rapamycin can also induce a selective autophagy-like process targeting EEFs in Hepa1-6. The rate of autophagic EEFs was 60–70% at 24 h after incubation with rapamycin, which was much higher than the rate seen in the control (*P* = 0.002, Fig. 2B). The administration of the autophagy inhibitor 3-MA (3-metholadine)^19^ reversed both non-selective autophagy by Hepa1–6 and the selective autophagy-like process targeting EEFs in rapamycin-induced Hepa1-6 (Fig. 2A,B).

The percentage of autophagic EEFs fusing with lysosomes was increased from less than 20% to approximately 70% when rapamycin was added (Fig. 2C). To monitor autophagy flux, CQ (chloroquine), which elevates the pH of lysosomes and blocks the fusion of autophagosomes with lysosomes^20^, was administered concurrently with rapamycin. We found that the administration of CQ significantly increased the rate of EEF autophagy, but not the generation of autolysosomes in the Hepa1-6 cells (Fig. 2B,C). Again, autophagy inhibitor 3-MA also reduced the rate of fusion of autophagic EEFs with lysosomes to the baseline level when Hepa1-6 cells were treated with rapamycin alone or with CQ (Fig. 2A,C). Therefore, our data strongly suggest that both non-selective autophagy and the selective autophagy-like process targeting EEFs in hepatocytes could be greatly enhanced by rapamycin.

### The effect of rapamycin-induced autophagy-like events on EEF development

**Figure 3:**
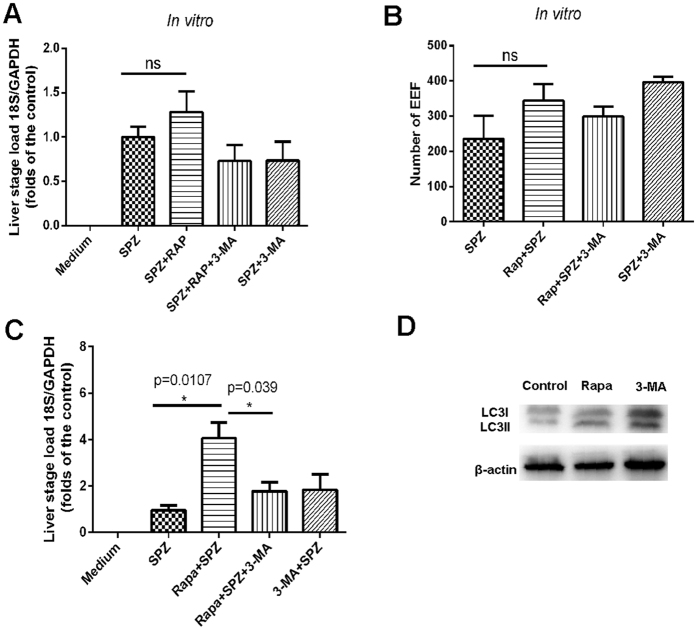
The effect of rapamycin-induced hepatic autophagy on liver-stage development. After *P. y. yoelii* 265BY or *P. y. yoelii* 265BY-RFP sporozoites were incubated with Hepa1-6 for 3 h, rapamycin, 3-MA or a combination of the two was then added. After 48 h, the cells were collected. (**A**) The ratio of parasite-specific 18 srRNA to mouse GAPDH was detected using real-time PCR. (**B**) The number of EEFs was counted under the fluorescence microscope. (**C**) The liver parasite load was detected at 42 h after the mice were challenged with sporozoites and treated with the indicated stimulus. (**D**) The levels of LC3I/II in cells treated with rapamycin or 3-MA were detected by Western blot (cropped blot was displayed). Three individual experiments were performed, and all data are presented as the mean ± SD. ns, no significance. *P < *0.05.*

To investigate the effect of the rapamycin-induced autophagy-like events on the development of the pre-erythrocytic stage, the growth of EEFs in Hepa1-6 cells treated or untreated with rapamycin was detected either by immunofluorescence microscopy of *P. y. yoelii* 265BY-RFP or by real-time PCR. As shown in Fig. 3A,B, both the number of EEFs and the expression of the *P. y. yoelii* 265BY-specific 18 s rRNA gene were slightly higher in treated cells compared to the controls, and the co-administration of 3-MA slightly reduced these factors. However, no significant differences were found.

The effect of the rapamycin-enhanced autophagy-like process targeting EEFs in the liver stage was also investigated using real-time PCR *in vivo*. The administration of rapamycin at 3 h post-sporozoite infection greatly elevated the parasite load in the liver, and 3-MA significantly reversed the effect of rapamycin (Fig. 3C). The inhibition of mTOR by rapamycin is far upstream in the autophagic process, and it is likely that other signalling events lead to compensation^21^. To confirm that the effect of rapamycin on EEF development was closely associated with its induction of an autophagy-like event, the LC3 I/II levels in Hepa1-6 treated with rapamycin or 3-MA were monitored. A Western blot showed that the ratio of the conversion of LC3I to LC3II could be significantly increased by rapamycin and reduced by 3-MA (Fig. 3D). Therefore, our data suggest that the induction of an autophagy-like event by rapamycin can promote liver-stage development.

### EEFs normally survive and replicate in the autolysosome

**Figure 4:**
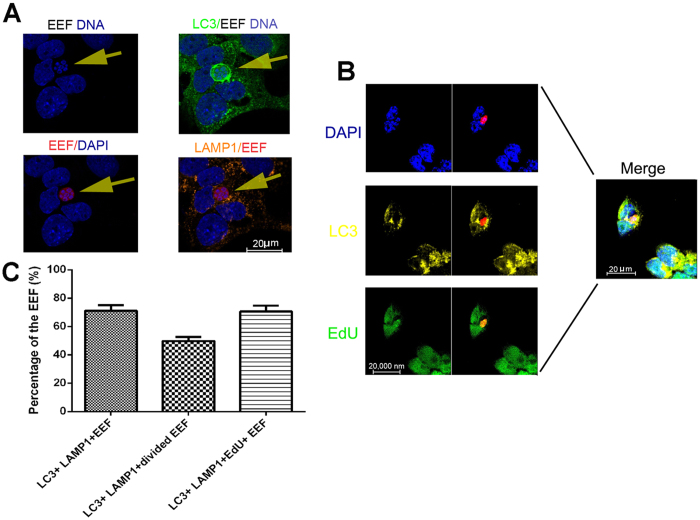
EEFs in the autophagosome survived and replicated normally. After Hep1-6 cells were incubated with *P. y. yoelii* 265BY-RFP for 24 h, the cells were stained with anti-LC3 and anti-LAMP1, which were followed by a DAPI counterstain or anti-LC3 and anti-EdU and a DAPI counterstain, and then subjected to confocal microscopy. (**A**) A representative confocal image of the survival of EEFs in the LC3 and LAMP1 double-positive vesicles. (**B**) A representative confocal image of the replication of EEFs in the autophagosome. (**C**) Quantification of EEFs surviving in the autophagosome. Three individual experiments were performed, and all data are presented as the mean ± SD. ns, no significance.

As mentioned above, rapamycin can augment both non-selective autophagy and a selective autophagy-like process targeting EEFs in hepatocytes. Although the non-selective autophagy of hepatocytes has been reported to promote liver-stage development by providing nutrients^15^, the effect of the selective autophagy-like process on EEF development is still largely unknown. Hepa1-6 cells were stained with LC3, along with either DAPI or EdU (5′ethynyl-2′ deoxyuridine), which is incorporated into replicating DNA, after the sporozoites were incubated with Hepa1-6 for 3 h and then treated or untreated with rapamycin for 24 h. DAPI staining showed that more than 65% nuclei of EEFs divided in the LC3 and LAMP1 double-positive vacuoles, indicating the survival and replication of EEFs in the autolysosome-like vesicle (Fig. 4A). This finding was further confirmed by EdU staining, which showed that EdU was incorporated into the Hepa1-6 nuclei, as well as into the EEF nuclei in all LC3-positive autophagosomes (Fig. 4B). Thus, these data suggest that EEFs can survive and replicate in autophagosomes treated with rapamycin.

### EEFs inhibit the maturation of autolysosomes

**Figure 5:**
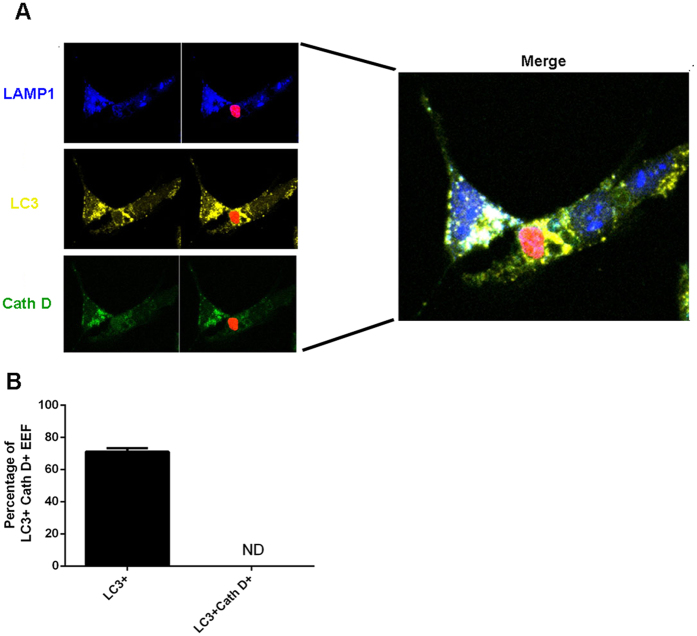
The maturation of EEF-containing autolysosomes was inhibited. After Hep1-6 cells were incubated with *P. y. yoelii* 265BY-RFP for 48 h, the cells were stained with anti-LC3, anti-LAMP1 and anti-cathepsin D, then subjected to confocal microscopy. (**A**) A representative confocal image of EEF in cathepsin D-negative autolysosome. (**B**) Quantification of EEF in cathepsin D-negative autolysosomes. Three individual experiments were performed, and all data are presented as the mean ± SD.

Surprisingly, EEFs can survive in autophagosomes, as most microorganisms are degraded in mature lysosomes. One possible interpretation is that EEFs inhibit the maturation of autolysosomes. Therefore, we investigated the maturation of autolysosome-containing EEFs by staining both lysosome marker LAMP1 and lysosomal proteolytic enzyme Cathepsin D 24 h after the incubation of sporozoites with Hep1-6 cells treated or untreated with rapamycin. As a result, all parasite-containing autolysosomes were both LC3- and LAMP1-positive, but none were stained with Cathepsin D (Fig. 5A,B), indicating that the malaria parasite inhibits autolysosome maturation. These data also strongly suggest that the survival and replication of EEFs might be closely associated with their ability to inhibit autolysosome maturation.

## Discussion

Non-selective autophagy by hepatocytes has been reported to promote the development of the malaria liver stage^15^, but the effect of selective autophagy on the fate of the parasite is still largely unclear. Here, we provide evidence that *P. y. yoelii* sporozoite infection induces a hepatic selective autophagy-like process targeting EEFs that is enhanced by rapamycin treatment. However, we found that EEFs in rapamycin-induced autolysosomes still survive and replicate and that the maturation of autolysosomes was likely inhibited by the EEFs. Thus, we present a novel escape strategy of the malaria liver stage.

Consistent with previous studies^15^,^16^, we found that sporozoite infection could also induce a selective autophagy-like process targeting EEFs in Hepa1-6 cells (Fig. 1A). However, we did not detect an accumulation of LC3 around the parasite at the very early stage (6 hpi) (Fig. 1A), a result that is somewhat different from previous findings that LC3 was observed surrounding the parasite at 1 hpi. The discrepancy might be explained by the fact that *P. y yoelii* was used in our study, while *P. berghei* was used in the previous studies^15^,^16^. Although no further investigation was performed in our study, other studies have indicated that autophagy of EEFs is a novel form of selective autophagy^15^. Both immunofluorescence microscopy and electronic microscopy have demonstrated that LC3 is deposited on the PVM (parasitophorous vacuole membrane), not in the autophagosome^15^,^16^,^22^.

Although several previous studies have reported that rapamycin slightly promotes EEF development^15^,^16^,^23^, it is not clear whether rapamycin promotes EEF development through the induction of autophagy. Here, we provide evidence that rapamycin enhances not only non-selective autophagy by host cells but also the selective autophagy-like process targeting EEFs (Fig. 2). Additionally, its effect on EEF development could be reversed by the autophagy-inhibitor 3-MA (Fig. 2A,C), which was closely associated with EEFs’ ability to regulate the activation of LC3 (Fig. 3D). Thus, we demonstrated that rapamycin promotes EEF development through the induction of autophagy-like events. As the inhibition of mTOR could increase the number of regulatory T cells and suppress host immune responses *in vivo*^24^, the observed rapamycin-enhanced liver-stage development *in vivo* might be overestimated.

The rapamycin-induced nonselective autophagy of hepatocytes could be one explanation for the observed rapamycin-enhanced development of the liver stage, as nonselective autophagy by hepatocytes facilitates EEF development by providing nutrients^15^. However, it is not sufficient to conclude that rapamycin enhances the development of the liver stage. Comparative genomic analysis has uncovered some, but not all, orthologues of ATG genes in the malaria genome^25^,^26^. Of these orthologues, ATG8 is expressed in all stages and has been implicated in apicoplast biogenesis of both *P. b* and *P. f *25,26. We found that treatment with rapamycin can also induce the transcription of *P. y. yoelii* ATG8 in the liver stage (Fig. S1A) and significantly promote the development of the *P. b* ANKA asexual stage (Fig. S1B). As the main mature enucleate RBCs in which the parasite replicates do not respond to rapamycin, our data suggest that rapamycin may have a direct effect on EEFs in the liver stage.

It is well known that apicomplexan protozoans such as *Plasmodium* and *Toxoplasma gondii* are always enclosed in a parasitophorous vacuole when they enter the host cell as an immune-evasion strategy to avoid degradation by the endocytic/lysosome system^27^. However, treatment with CD40 could induce the fusion of parasitophorous vacuoles of *Toxoplasma gondii* with late endosomes/lysosomes in macrophages by triggering autophagy^28^. We found that rapamycin could promote the fusion of EEF-containing autophagosomes with lysosomes (Fig. 2A). However, some EEFs in the autolysosomes could still survive and replicate (Fig. 4A), a finding consistent with a recent study showing that some parasites could still survive following selective autophagy by the host cell^15^. However, some EEFs were cleared by selective autophagy. A further study showing that parasite survival following the selective autophagy-like process is closely associated with inhibition of autolysosome acidification by EEFs, although the mechanism underlying this process is still largely unknown (Fig. 5A). Therefore, the survival of EEFs in autolysosomes might be a basic requirement for rapamycin-associated enhancement of liver-stage development.

Although most evidence suggests that selective autophagy could restrict the growth of some bacteria and viruses^29^, ^30^, ^31^, ^32^, ^33^, some microorganisms have developed strategies to evade host autophagy. For example, *Mycobacterium tuberculosis, Legionella pneumophila* and *Yersinia pestis* evade host autophagy by inhibiting its initiation^34^, directly interfering with host components involved in autophagy^35^, or blocking the fusion of the phagosome with the lysosome^36^. Our findings show that EEFs also inhibit autolysosome maturation by an unknown mechanism, indicating that this pathway might be an important escape strategy.

In conclusion, we reported that *P. y. yoelii* sporozoite infection could induce the selective autophagy-like process targeting EEFs, which can be enhanced by rapamycin treatment. However, EEFs can survive in hepatocyte autolysosomes by inhibiting their maturation via an unknown mechanism, which might be a novel strategy of autophagy escape in microorganisms.

## Materials and Methods

### Plasmodium and mice

*P. y. yoelii* 265BY-RFP was constructed by inserting RFP into the ssurRNA gene of *P. y. yoelii* 265BY. In brief, PL1102 (a gift from MR4) was electroporated into cultured shizonts of *P. y. yoelii* 265BY and injected into a mouse. One day later, the mice were treated with pyrimethamine in their drinking water for six days and parasites were collected and cloned. Clones of *P. y. yoelii* 265BY-RFP were identified by both PCR and fluorescence microscopy, as previously described^37^. Both *P. y. yoelii* 265BY and *P. y. yoelii* 265BY-RFP were maintained by alternate passaging between the mosquitoes and Kunming mice, which were purchased from the Experimental Animal Center of the Third Military Medical University. All methods were carried out in accordance with the Guide for the Care and Use of Laboratory Animals of the Third Military Medical University. All protocols were approved by the Animal Institute of the Third Military Medical University.

### Mosquito rearing and infection

*Anopheles stephensi* (Hor strain) were fed with a 5% sugar solution at 27 °C and 70–80% humidity. For infection, three- to five-day-old female adults were fed on *P. y. yoelii* 265BY- or *P. y yoelii* 265BY-RFP-infected Kunming mice at 23–24 °C, with gametocytemia up to 0.5%. Seven days after the blood meal, the mosquitoes were dissected and oocysts in the midgut were examined under a fluorescence microscope.

### Sporozoites purification

At 17 days after infection with *P. y. yoelii* 265BY or *P. y. yoelii* 265BY-RFP, female mosquitoes were anesthetised and washed in 75% ethanol to remove any surface contamination. Their salivary glands were dissected and collected in DMEM containing 100 units/ml penicillin and 100 μg/ml streptomycin. The mosquitoes were extensively washed in the DMEM. The salivary glands were dissected and ground in a 1.5 ml tube, and the released sporozoites were purified by DEAE cellulose chromatography, as previously described^38^.

### Incubation of sporozoites with Hepa1-6 cells

Hepa1-6 cells were purchased from ATCC and kept in DMEM supplemented with 10% FBS (Hyclone), 1% penicillin/streptomycin and 1% L-glutamine (Invitrogen). For infection, 1 × 10^5^ cells were seeded on coverslips in 24-well plates and incubated with 2 × 10^5^ purified *P. y. yoelii* 265By-RFP sporozoites for 3 h. Cells were then washed three times with DMEM containing 1% penicillin/streptomycin to remove the extracellular sporozoites. The samples were then treated with or without 270 μM of rapamycin (Dissolved in DMSO and then diluted by PBS), 10 mM 3-MA (sigma), rapamycin plus 40 μM CQ (chloroquine), rapamycin plus 3-MA, or a combination of the three.

### Immunofluorescence and confocal microscopy

After Hepa1-6 cells growing on the coverslip were incubated with *P. y. yoelii* 265BY-RFP sporozoites for the indicated time, the cells were fixed in 4% paraformaldehyde for 15 min at room temperature, permeabilised with ice-cold methanol at −20 °C for 10 min, and blocked in 5% BSA and 0.3% TritonX-100/PBS for 1 h at room temperature. To detect autophagy, the cells were then stained with anti-LC3 (Abcam) and anti-LAMP1 (Abcam). They were then stained with Dylight 488 or Dylight 405-labeled secondary antibodies (Abcam), respectively. To investige the survival and replication of EEFs in the autolysosomes, the cells were stained with anti-LC3 (Abcam), anti-LAMP1 (Abcam) and anti-EdU (Abcam). To detect autolysosome maturation, the cells were stained with anti-LC3 (Abcam), anti-LAMP1 (Abcam) and Anti-Cathepsin D (Abcam), then, stained with Dylight 488, Dylight 405 or Dylight 649-labeled secondary antibody (Abcam), respectively. DNA was counterstained with DAPI in all of the above experiments. Coverslips were mounted with Dako Fluorescent Mounting Medium (Dako), and all images were acquired on a Leica DM 2000 confocal microscope and analysed using the Leica Application suite, version 2.3.0.

### Western blot

Hepa 1-6 cells were cultured in the presence or absence of 270 μM rapamycin or 10 mM 3-MA. After 24 h, cells were collected and lysed in SDS lysis buffer (Beyotime). The lysate was separated by SDS-PAGE and transferred to PVDF (polyvinylidene difluoride) filters. The filters were immunoblotted using anti-LC3 (Abcam) and anti-β-actin polyantibody (Sigma) and then developed with ELC (Pierce).

### Taqman Real-time PCR

The parasite load was determined by the detection of plasmodium-specific 18 S rRNA using real-time PCR, as previously described^39^. To detect parasite load *in vitro*, cells were collected 48 h after incubation of the sporozoites with Hepa1-6 cells in the presence or absence of rapamycin, 3-MA or both. To detect liver parasite load, each mouse was injected *i.v.* with 1000 sporozoites and then injected *i.p.* with 2 mg/kg rapamycin or with 15 mg/kg 3-MA 2 h later. The liver was dissected 42 h post-challenge. Total RNA from the cells or liver was isolated using Trizol (Invitrogen) and reverse-transcribed using random primers. Then, real-time PCR reactions for 18 S rRNA and GAPDH were carried out with Premix Ex Taq (GeneCore BioTechnologies Co, Vita Genomics, Inc.). Real-time PCR was performed in the Eco (Illumina, San Diego, USA, Inc.), and the parasite load of each sample was expressed as the ratio of parasite 18 S rRNA to mouse GAPDH.

### Statistical analysis

All data were analysed with an unpaired *t* test using Graphpad prism version 5.0. *P* < *0.05* was considered statistically significant.

## Additional Information

**How to cite this article**: Zhao, C. *et al*. The rodent malaria liver stage survives in the rapamycin-induced autophagosome of infected Hepa1–6 cells. *Sci. Rep.* **6**, 38170; doi: 10.1038/srep38170 (2016).

**Publisher's note:** Springer Nature remains neutral with regard to jurisdictional claims in published maps and institutional affiliations.

## Supplementary Material

Supplementary Information
