# Supplementary material for: The rodent malaria liver stage survives in the rapamycin-induced autophagosome of infected Hepa1–6 cells
Source: Sci Rep. 2016 Nov 30;6:38170. doi: 10.1038/srep38170 (PMC5128998; doi:10.1038/srep38170)
Supplement: Supplementary Information [file srep38170-s1.pdf]

The rodent malaria liver stage survives in the rapamycin-induced autophagosome of infected Hepa1-6 cells

Chenghao Zhao[1#2#], Taiping Liu[1#], Taoli Zhou[1], Yong Fu[1], Hong Zheng[1], Yan Ding[1], Kun Zhang[1],Wenyue Xu[1*]

Material and Methods

SYBR quantitative PCR assay for detection of P.y yoelii ATG8

After 2×10^5 *P.y yeolii* sporozoites were incubated with 1×10^5 Hepa 1-6 for 3 h, cells were washed to remove the extracellular sporozoites, and then treated with or without rapamycin, 3-MA, or their combination for 24 h. Total RNA was isolated and reversely transcribed, and the PCR reaction was set up using SYBR ® Premix Ex Taq TM II 2× (TAKARA, Japan). The PCR conditions consisted of an initial denaturation at 95°C for 30 s followed by amplification for 40 cycles of 15 s at 95℃ and 50 s at 60℃. The primers for *P.y yoelii* ATG8 were , the levels of *P. y yoelii* ATG8 and mouse GAPDH were measured semi-quantitatively using Illumina Eco software (Illumina, San Diego, USA, In). The relative level of ATG8 was expressed the ratio of *P. y yoelii* ATG8 to mouse GAPDH.

Effect of rapamycin on P.b ANKA asexual stage development

Blood was taken from the *P.b* ANKA-infected mouse by cardiac puncture, and then centrifuged at 2000 rpm for 3 min to remove the serum. The pellet was suspended in RPMI 1640 complete medium with or without rapamycin, and then cultured in flask with 5% CO2, 5% O2 and 90% N2. 16 h later, *P.b* ANKA pRBCs were collected and stained with Giemsa, and parasitemia was quantificated.

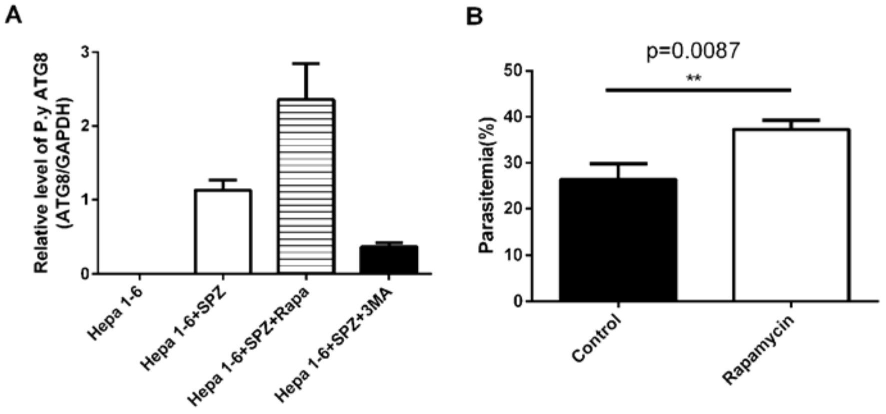

Figure S1. The effect of rapamycin on the transcription of *P. y yoelii* ATG8 and *P.b* ANKA asexual stage development. A, Sporozoites were incubated with Hepa 1-6 and treated with or without rapamycin, 3-MA, or their combination for 24 h. Total RNA was isolated and reversely transcribed, and the levels of both *P. y yoelii* ATG8 and mouse GAPDH were detected by real-time PCR. The relative level of *P. y yoelii* ATG8 was expressed as the ratio of *P. y yoelii* ATG8/GAPDH. B, *P.b* ANKA pRBCs was taken from the infected mouse, and cultured in the presence or absence of rapamycin for 16 h. *P.b* ANKA pRBCs were stained with Giemsa, and parasitemia was quantified.
